# Cowpea Nodules Harbor Non-rhizobial Bacterial Communities that Are Shaped by Soil Type Rather than Plant Genotype

**DOI:** 10.3389/fpls.2016.02064

**Published:** 2017-01-20

**Authors:** Jakson Leite, Doreen Fischer, Luc F. M. Rouws, Paulo I. Fernandes-Júnior, Andreas Hofmann, Susanne Kublik, Michael Schloter, Gustavo R. Xavier, Viviane Radl

**Affiliations:** ^1^Soil Science Department, Universidade Federal Rural do Rio de JaneiroSeropédica, Brazil; ^2^Embrapa AgrobiologiaSeropédica, Brazil; ^3^Research Unit Environmental Genomics, Helmholtz Zentrum MünchenOberschleißheim, Germany; ^4^Embrapa SemiáridoPetrolina, Brazil

**Keywords:** *Vigna unguiculata* (L.) Walp, endophytes, microbiome, *Bradyrhizobium*, *Chryseobacterium*

## Abstract

Many studies have been pointing to a high diversity of bacteria associated to legume root nodules. Even though most of these bacteria do not form nodules with legumes themselves, it was shown that they might enter infection threads when co-inoculated with rhizobial strains. The aim of this work was to describe the diversity of bacterial communities associated with cowpea (*Vigna unguiculata* L. Walp) root nodules using 16S rRNA gene amplicon sequencing, regarding the factors plant genotype and soil type. As expected, *Bradyrhizobium* was the most abundant genus of the detected genera. Furthermore, we found a high bacterial diversity associated to cowpea nodules; OTUs related to the genera *Enterobacter, Chryseobacterium, Sphingobacterium*, and unclassified *Enterobacteriacea* were the most abundant. The presence of these groups was significantly influenced by the soil type and, to a lesser extent, plant genotype. Interestingly, OTUs assigned to *Chryseobacterium* were highly abundant, particularly in samples obtained from an Ultisol soil. We confirmed their presence in root nodules and assessed their diversity using a target isolation approach. Though their functional role still needs to be addressed, we postulate that *Chryseobacterium* strains might help cowpea plant to cope with salt stress in semi-arid regions.

## Introduction

Many leguminous plants are able to establish symbiosis with a variety of bacterial species of the phylum Proteobacteria, which are collectively referred to as rhizobia. This symbiosis is characterized by the formation of nodules on the roots and/or stems of plants, where the bacteria encounter optimal conditions for biological nitrogen fixation (BNF; Graham, 2008). The formation of these nodules is a highly regulated process, the success of which depends on the exchange of specific signaling molecules between the micro and the macrosymbiont (Oldroyd et al., 2011). Bacteria have been isolated from legumes nodules since 1888 (Frank, 1889; Beijerinck, 1890), resulting in more than a hundred described species (Peix et al., 2015). Bacteria capable of inducing nitrogen-fixing nodules on the roots of leguminous plants are found among the Alphaproteobacteria (e.g., *Rhizobium, Bradyrhizobium, Ochrobactrum, Ensifer, Phyllobacterium, Mesorhizobium, Devosia, Azorhizobium, Allorhizobium, Shinella, Methylobacterium*, and *Microvirga*) and Betaproteobacteria (genera *Burkholderia* and *Cupriavidus*; Gyaneshwar et al., 2011; Remigi et al., 2016).

In the past it was assumed that the interior of root nodules are exclusively colonized by rhizobial strains. However, recent studies have been pointing to a much higher diversity of bacteria associated to root nodules (De Meyer et al., 2015). Several studies on rhizobial diversity using standard cultivation methods (Vincent, 1970) detected non-rhizobial endophytes (NRE) in nodules of different legumes species. They belong to many different genera including *Agrobacterium, Arthrobacter, Acinetobacter, Bacillus, Bosea, Enterobacter, Micromonospora, Mycobacterium, Paenibacillus, Pseudomonas*, and *Stenotrophomonas* (Velázquez et al., 2013). Even though those strains do not induce nodule formation, they might be able to enter infection threads when co-inoculated with rhizobial strains (Pandya et al., 2013; Zgadzaj et al., 2015). NRE include potential plant growth promoting rhizobacteria (PGPR), as some of the investigated strains display PGPR traits, such as indole acetic acid production or phosphate solubilization (Li et al., 2012), or enhance nodulation when co-inoculated with a rhizobial symbiont compatible with the host plant (Martínez-Hidalgo et al., 2014). Nevertheless, the role of NRE present in the nodules remains mostly unknown. Interactions between endophytes and hosts are very complex and might vary from mutualistic to antagonistic, depending on the environmental conditions (Partida-Martinez and Heil, 2011).

The overwhelming effect of the soil on the setup of the rhizosphere bacterial communities has been demonstrated in many studies (Marschner et al., 2001; Girvan et al., 2003; Berg and Smalla, 2009). Even for bacteria living inside or strongly adhered to root tissue and, hence, probably submitted to analogous selective pressure, it was shown that the soil significantly influences community composition (Bulgarelli et al., 2012). We hypothesized that the composition of NREs is more influenced by the native soil microbial community than by the plant genotype. Moreover, this should not apply to rhizobial bacterial symbionts, for which the infection is regulated by a highly specific signal exchange between host and symbionts (Kobayashi and Broughton, 2008; Oldroyd et al., 2011). To address those questions, we performed field trials in two soils with different textures, where we cultivated two different genotypes of cowpea (*Vigna unguiculata* L. Walp), a major legume crop in semi-arid regions of Africa, South America and Asia. In those trials, we evaluated the diversity of bacteria inside the nodules by 16S amplicon sequencing and isolated the major NRE genus detected in the libraries.

## Materials and Methods

### Field Experiment

For the investigation of the diversity of bacterial communities associated to root nodules, cowpea was grown in two experimental stations of the Embrapa Semiárido located at the Northeast of Brazil, in a semi-arid climate zone. The areas were chosen mainly due to their differences in soil characteristics. At the Mandacaru experimental field {(MEF) municipality of Juazeiro, State of Bahia: 09°24′S 40°26′W} cowpea was grown in a silty clay Haplic Vertisol. At the Bebedouro experimental field {(BEF) municipality of Petrolina, State of Pernambuco: 09°09′S 40°22′W} the soil was characterized as a sandy loam Red Yellow Dystrophic Ultisol. Both sites had no history of cowpea cultivation and were previously cultivated with annual crops. Before cowpea was sowed, the MEF and BEF sites had been cropped with maize and watermelon, respectively. The physico-chemical analysis of the top soil of the stations is shown in **Table 1**. Soils were fertilized with 20 kg of P_2_O_5_ ha^-1^ and 20 kg of K_2_O ha^-1^. We chose the cultivars BRS Acauã and BRS Pujante (Embrapa, Brazil) which were specifically bred for this climatic region. Uninoculated seeds were sown in May 2013. A completely randomized block design was used with four replications per cultivar, resulting in eight plots (5m × 4m) per area. Each plot was composed by eight rows (5 m long) spaced by a distance of 0.5 and 0.2 m between plants in the rows.

**Table 1:** Soil chemical characteristic of the top soil of the stations is shown.

Soils type	Haplic Vertisol	Red yellow dystrophic Ultisol
Organic matter (g.kg^-1^)	7.2	6.3
pH (H_2_O)	6.8	6.3
P (mg.dm^3^)	44.62	11.92
K (cmolc.dm^-3^)	0.36	0.33
Ca (cmolc.dm^-3^)	20.4	2.0
Mg^2+^ (cmolc.dm^-3^)	5.6	0.4
Al^3+^ (cmolc.dm^-3^)	0.05	0.05
H+Al^3+^ (cmolc.dm^-3^)	4.62	0.66
Some of bases – S (cmolc.dm^-3^)	26.45	2.78
Cation exchange capacity – CEC (cmolc.dm^-3^)	31.07	3.44
Base saturation – V (%)	85	81

Samples were taken during flowering, at 35 days after emergence. From each plot four plants were collected. We analyzed one composite sample per plot, which was formed by 80 nodules taken from the four plants (20 per plant). For surface sterilization, nodules were treated with 70% ethanol for 1 min, followed by 2% hypochlorite for 5 min and washed six times with sterile distilled water. The efficiency of the sterilization was checked by plating 100 μl from the water of the last washing step on nutrient agar plates incubated for 72 h at 28°C. Surface sterilized nodule samples were kept at -20°C until further analysis.

### DNA Extraction from Nodule Samples

For DNA extraction, 20 nodules of each composite sample (described above) were frozen in liquid nitrogen and ground with sterile mortars and pestles. One gram of macerated nodules (2× 0.5 g) was extracted according to Töwe et al. (2011). The quality and quantity of the DNA extracts were evaluated in 1% agarose gels and using the PicoGreen dsDNA quantification assay (Invitrogen, USA), respectively.

### 16S rRNA Gene Amplification, Library Preparation, and Pyrosequencing

High-throughput sequencing was performed on a second-generation pyrosequencer (454 GS FLX Titanium, Roche, Germany). We used tagged-primers with Multiplex Identifiers (MIDs; Roche, Germany) for the PCR reaction. Amplification was performed using the primers 27 F (5′ AGA GTT TGA TCM TGG CTC 3′, Wilson et al., 1990) and 984 R (5′GTA AGG TTC YTC GCG 3′, Klindworth et al., 2013). Reaction mixtures contained 1.25 U FastStart Taq DNA Polymerase (Roche, Germany), 10 pmol of each primer (Metabion, Germany), 200 μM dNTPs (Roche, Germany), 0.3% bovine serum albumin (Sigma-Aldrich, Germany) and 5 ng DNA template to a final volume 25 μl. PCR reaction conditions were as follows: 95°C for 5 min; 30 cycles of denaturation at 94°C for 1 min, annealing at 50°C for 1 min, and extension at 72°C for 1 min; followed by final elongation at 72°C for 10 min. All samples were purified using the NucleoSpin^®^ Gel and PCR Clean-up kit (Macherey-Nagel, Germany) according to manufacturer’s instructions. The quality and quantity of the amplicon libraries were evaluated using 2% agarose gels and Quant-iT^TM^ PicoGreen^®^ dsDNA Assay Kit (Life technologies, Germany). The average fragment size of the amplicon libraries was measured with an Agilent 2100 bioanalyzer instrument using the Agilent DNA 7500 Kit (Agilent Technologies, Germany).

### Data Analysis

The automatic amplicon pipeline of the GS Run Processor (Roche, Germany) was used to perform an initial quality filtering of the pyrosequencing raw reads to remove failed reads, low quality reads and adaptor sequences. Sequences were processed for further analyses and quality checking using the Mothur software package version 1.33.3 (Schloss et al., 2009) and subjected to denoising (PyroNoise implemented in Mothur, Quince et al., 2009), clustering and data evaluation. Sequences shorter than 200 nt, with more than 7 nt long homopolymers or chimeras were removed. The remaining sequences were aligned using SILVA-based bacterial reference alignment^1^ (Release 119; Pruesse et al., 2007), with confidence threshold of 80%, to obtain the best taxonomic classification. OTUs were clustered with similarity of 97%, as it is the narrowest clustering distance recommended for 454 sequences (Kunin et al., 2010) as well as with similarity of 95 and 90%. OTUs occurring only one time in the dataset (singletons) were excluded from the evaluation to avoid overestimation of the diversity. For diversity analyses equal sample effort is required. Therefore, all samples were subsampled to the number that corresponded to the one obtained for the library with the smallest number of reads. The subsampled datasets were submitted to rarefaction analyses at OTU similarity levels of 97, 95, and 90%. Statistics with the subsampled dataset was performed using the software R-project version R 2.15.1^2^ (R Core Team, 2014) with RStudio [version 0.98.1062^3^ and the packages MASS (Venables and Ripley, 2002), gplots (Warnes et al., 2012), and vegan (Oksanen et al., 2012)]. The R software package was used to calculate ANOVA and multiple comparisons with adjusted *p*-values including Bonferroni tests. Phylogenetic trees based on representative sequences of OTUs as well as sequences from the isolated bacteria were generated using the ARB software package (Ludwig et al., 2004) using the All-Species Living Tree Project (Yarza et al., 2008) as basis for calculation. Sequence files were deposited in the NCBI Sequence Read Archive^4^ under the accession numbers SAMN05437328–SAMN05437342.

### Isolation of *Chryseobacterium* Associated to Cowpea Nodules

To confirm the presence of living cells from one of the major groups detected by the 16S rRNA gene amplicon analyses, we used a target isolation approach described by Nishioka et al. (2016). The cowpea genotypes BRS Pujante and BRS Acauã were sown in BEF in June, 2016 using the same experimental design as described above, except for the number of plots (*n* = 3 per genotype). Sampling was done at the flowering stage. Four plants were taken per plot and mixed to form a composite sample, resulting in three replicates per cultivar. The roots were separated from the shoots, washed with tap water to remove all adhered soil, transferred to plastic bags and stored at 10°C until further isolation procedures. Nodules were then detached from the roots, surface sterilized using 3% hypochlorite for 5 min, and washed 10 times in sterile distilled H_2_O. This sterilization method has been previously evaluated by rolling the nodules over agar plates. Furthermore, the water used in the last washing step was spread on R2A plates. After sterilization, 10 healthy, undamaged nodules were randomly chosen per sample. Each nodule was crushed in 2 ml tubes with 1 ml sterile 0.85% NaCl using sterile pestles. One hundred microliter of the extracts were spread in R2A agar supplemented with 50 μg ml^-1^ cycloheximide and 1 μg ml^-1^ tobramycin. After 4 days of incubation at 28°C, characteristic colonies were purified by streaking on R2A agar plates.

### Amplification and Sequencing of 16S rRNA Genes from Bacterial Isolates

To obtain genomic DNA, bacterial isolates were grown for 2 days in R2A broth. DNA extraction was carried out with the commercial kit Wizard Genomic DNA purification System (Promega, USA) according to the manufacturer’s instructions. The 16S rRNA genes of the isolated strains were amplified by PCR using the GoTaq Flexi kit (Promega). Reactions occurred in 50 μl volumes containing 1.25 U GoTaq Flexi DNA Polymerase (Promega), 10 pmol of each primer (Alpha DNA, Montreal, Canada), 200 μM dNTPs (Invitrogen), 1.5 mM MgCl_2_ and 50 ng DNA template. PCR reaction conditions were as follows: 95°C for 5 min; 30 cycles of denaturation at 94°C for 1 min, annealing at 50°C for 1 min, and extension at 72°C for 1 min; followed by final elongation at 72°C for 10 min and the primers 27F (5′ AGA GTT TGA TCM TGG CTC 3′) and 1492R (3′- TAC CTT GTT ACG ACT T - 5′; Wilson et al., 1990; Lane, 1991). Amplicons were partially sequenced with the 27F primer in a 3500 Genetic Analyzer device (Applied Biosystems), using the BigDye Terminator v3.1 Cycle Sequencing kit following the manufacturer’s instructions. Comparative analyses of the obtained sequences and close relatives found in the database were performed using the ARB software package^5^ (Ludwig et al., 2004) with the All-Species Living Tree Project (Yarza et al., 2008) as basis for calculation. Sequences were deposited in the GenBank under the accession numbers KX907716–KX907726.

## Results

### Basic Characteristics of the Amplicon Libraries

We evaluated the microbiome of cowpea root nodules in two different soil types and two cultivars at the stage of flowering using 16S rRNA gene amplicon sequencing. We obtained 1040–11528 reads per sample with an average of 6489 reads. After equalizing the sampling effort, 16640 reads were further analyzed and subsampling for statistical analyses was performed using 1040 reads per samples. When the threshold was set at 97% similarity and singletons were excluded, we obtained, overall, 707 OTUs. On average 87 ± 19 OTUs were detected per sample, indicating that only a minor part of the OTUs were shared among libraries from the different treatments. Rarefaction analyses did not indicate complete saturation at 97% similarity (**Supplementary Figure S1**), thus further analyses were performed at 95% similarity, for which, on average, coverages of 95% ± 1 were reached (**Table 2**). At this level 99 different OTUs were obtained after the filtering of the reads (Supplementary Table 1).

**Table 2:** Richness estimates, alpha diversity, and evenness based on OTUs clustered at 95% similarity level.

Source	Coverage	Chao	Shannon	
			Diversity (*D*)	Evenness (*E*_D_)
BRS Acauã Ultisol	96.0 (±1.0)	143.0 (±17.3)	0.28 (±0.04)	0.05 (±0.01)
BRS Acauã Vertisol	95.0 (±1.0)	174,2 (±19.7)	0.14 (±0.06)	0.10 (±0.05)
BRS Pujante Ultisol	95.0 (±1.0)	202.4 (±42.2)	0.22 (±0.11)	0.06 (±0.02)
BRS Pujante Vertisol	95.0 (±1.0)	200.9 (±53.0)	0.19 (±0.05)	0.06 (±0.02)

*Source = cowpea nodule samples collected from different plant genotypes and soil types.*

Based on these data, we calculated the community richness, diversity and evenness of nodule samples taken from the plant genotypes BRS Acauã and BRS Pujante grown in the two different soil types (**Table 2**). Similar values (*p* > 0.05) were obtained for all measured parameters, indicating that the diversity of bacterial communities associated to cowpea nodules was not influenced by soil type or plant genotype, at least at the similarity level of 95%.

### OTUs Assigned to Rhizobia

**FIGURE 1:**
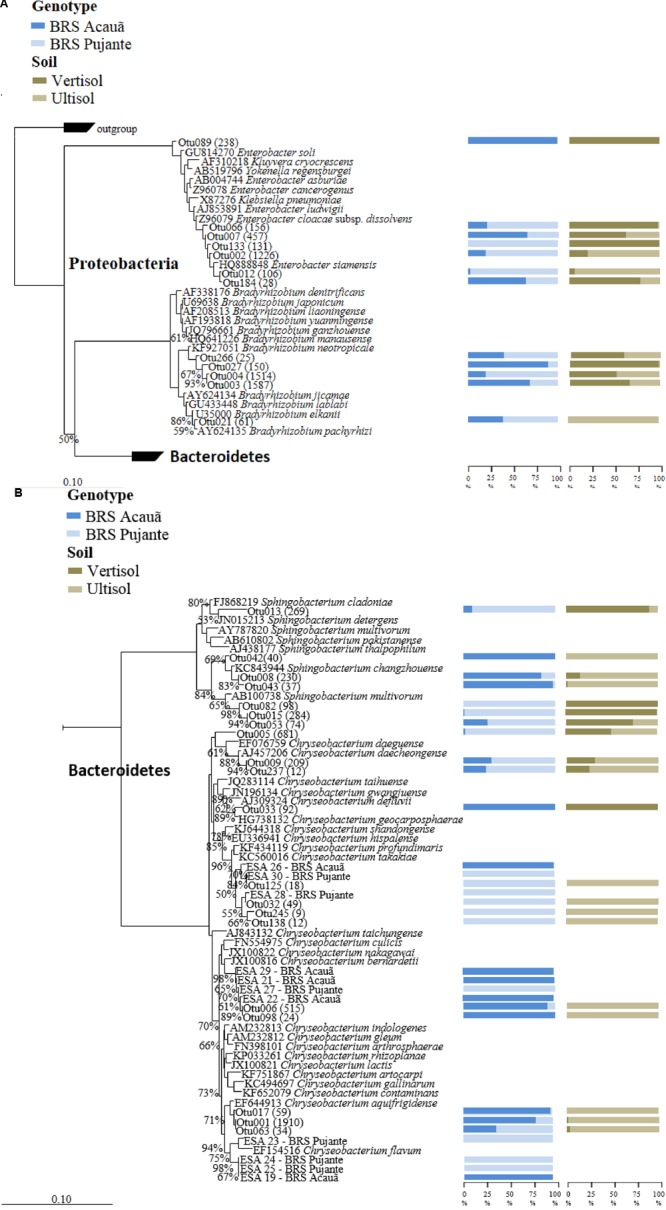
**Phylogenetic affiliation of major OTUs (A**: Proteobacteria) as well as the OTUs and isolates of *Chryseobacterium* (**B**: *Sphingobacterium* and *Chryseobacterium*) found in the 16S rRNA amplicon libraries. The phylogenetic trees were calculated with the neighbor joining method (Saitou and Nei, 1987). All sequences from valid described bacterial species are derived from type strains. Bootstrap values were inferred from 1000 replicates and are indicated at tree branches when ≥50%. The trees are drawn to scale, with branch lengths in the same units. The bars represent 10 estimated substitution per 1000 nucleotide positions. Blue (dark and light) and brown (dark and light) bars indicate the percentage of the OTU occurrence from cowpea genotype and soil, respectively. Number of reads for each OTU is given in parentheses. All isolates are identified as ESA and the genotypes they were detected (Acauã and Pujante).

We filtered the sequences of “true rhizobial symbionts,” here defined as bacteria that were previously demonstrated to induce nodule formation. As expected, sequences assigned to *Bradyrhizobium* spp. were detected in high abundances in all libraries, contributing, on average, to 25% of the total reads. OTU003 and OTU004, which formed a cluster with *Bradyrhizobium neotropicale*, were the only OTUs detected in all libraries, independent of soil or cultivar type (**Figure 1A**). Interestingly, we observed an effect of the plant genotype on their relative abundance, being OTU003 and OTU004 the most predominant for the genotypes BRS Acauã and BRS Pujante, respectively. Furthermore, we observed higher abundances of OTU003 in the Vertisol, indicating a combined effect of plant and soil type. In addition, the ubiquitous, but less predominant OTU027 and OTU266 are also included in this cluster with *B. neotropicale*. Another, less abundant, OTU closely related to *B. pachyrhizi* and *B. elkanii*, was more influenced by soil type than the predominant OTUs, being detected only in the Ultisol samples. No other putative rhizobial sequences were detected in the libraries, after quality check and filtering of the sequences.

### Nodule Associated Non-rhizobial OTUs

**FIGURE 2:**
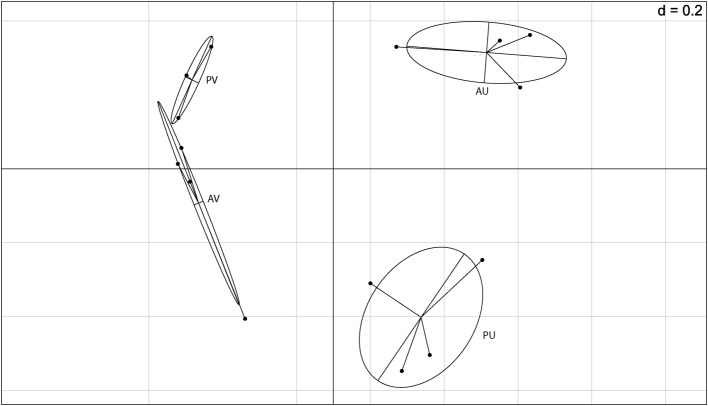
**Between group analysis (BGA) out of principal component analysis (PCA) based on 16S rRNA barcoding sequence data (OTU 5% dissimilarity).** A = BRS Acauã and P = BRS Pujante, U = Ultisol and V = Vertisol.

After filtering the putative rhizobial OTUs, we visualized the Euclidean distances of the remaining OTUs using principal component analysis and between group analyses (BGA) to determine if the composition of the NREs is influenced by soil type and/or plant genotype (**Figure 2**). Although for one of the areas, we observed grouping based on plant genotype, the soil was the major driver of composition of bacterial communities associated to the nodules. A PERMANOVA test was carried out to test the significance of the groupings. The soil type significantly affected the bacterial community composition (*p* = 0.001) found in the cowpea nodules. Even though this was not verified for plant genotype (*p* = 0.193), we detected a significant combined effect of soil and plant genotype (*p* = 0.042).

The OTUs were aligned with the SILVA database for classification. OTUs detected in nodule samples were assigned to the phyla Proteobacteria, Bacteroidetes, Actinobacteria, and Firmicutes, although the last phylum was only detected in the Ultisol samples and at very low abundance. As expected, Proteobacteria was the most predominant phylum. In general, Gammaproteobacteria and Alphaproteobacteria were the most abundant classes among the Proteobacteria and represented in average 29 and 25% of the reads, respectively. Moreover, we found differences in bacterial community composition at the class level, as Flavobacteria and Actinobacteria were mainly present in nodules collected from the Ultisol and the Vertisol, respectively.

**FIGURE 3:**
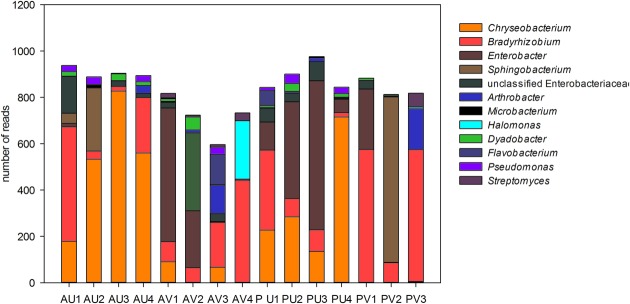
**Relative abundance (in total number of reads) of the bacterial genera present in the nodules of cowpea genotypes cultivated in different soil.** Only genera that represented more than 1% of the total reads are shown.

Analyses of the data at genus level gave a much more complex picture of bacterial communities associated to nodules. In total, 25 genera were observed in the libraries. From those, *Enterobacter, Chryseobacterium, Sphingobacterium*, and unclassified Enterobacteriaceae made up approximately 60% of the reads (**Figure 3**). Although some Enterobacteriaceae OTUs were highly abundant (see also **Figure 1A**), such as OTU002, none was ubiquitous and in most of the cases those were found only in a few libraries. Moreover, neither plant genotype nor soil type had clear effects on their presence. For the genus *Chryseobacterium*, we observed differences in OTU composition related to soil type. In general, the OTUs were related to five different clusters within the genus. The abundance of *Chrysobacterium***-**OTUs was higher in the Ultisol compared to the Vertisol. Two clusters, one related to *C. daeguense* and *C. daecheongense* (cluster I) and the other related to *C. aquifrigidense* and *C. flavum* (cluster II), contained OTUs detected in both soil types. OTU001, which had the highest number of reads of all OTUs, clustered with *C. aquifrigidense* and was mainly detected in the Ultisol samples (Cluster IV). The other two clusters exclusively contained OTUs found in Ultisol samples and are related to *C. profundimaris* and *C. takakiae* as well as *C. bernardetii*, respectively. OTU033 formed a cluster with *C. geocarposphaerae* and was exclusively found in Vertisol. Furthermore, other low abundant OTUs affiliated to the genera *Chitinophaga, Roseateles, Cronobacter*, and *Bacillus* were identified only in nodules of the Ultisol samples. On the other hand, *Halomonas, Streptomyces*, and *Nocardioides* were exclusively found in nodules from Vertisol. No clear presence pattern was observed for the plant genotype.

### NRE Isolated from Cowpea Nodules

In total, 29 characteristic yellow/orange colonies were detected on plates after inoculation with nodule extracts from both cowpea cultivars. Phylogenetic analyses based on 16S rRNA sequences showed that the 11 strains were affiliated to three previously described phylotypes within the genus *Chryseobacterium* (**Figure 1B**). Interestingly, we found the strains ESA 26, 28 and 30 were mainly detected in nodules of the genotype Pujante, as also observed for libraries (Cluster III). Similar results were obtained for strains ESA 21, 22, 27 (cluster IV) and 29, and ESA 19, 23, 24 and 25 (cluster V). Interestingly, eight other isolates were identified as Actinobacteria, most of them belonging to the genus *Microbacterium* (data not shown).

## Discussion

In the present study, we aimed to investigate the diversity of bacterial communities associated to cowpea nodules using a cultivation independent approach. It cannot be completely excluded that, besides true endophytes, also the DNA of some bacteria tightly attached to the surface of the nodules remains stable after the surface sterilization procedure. Therefore, to avoid misconception, we referred here to associated bacteria whenever DNA based methods were used to analyze the bacterial communities present in the nodules.

The 16S rRNA amplicon analyses of DNA extracts from surface sterilized root nodules detected 99 OTUs at 5% dissimilarity level. These OTUs were assigned to different genera within the Proteobacteria, Bacteroidetes, Actinobacteria, and Firmicutes phyla. Studies based on cultivation carried out in Africa and Asia, which are considered to be the origin of cowpea diversity, reported only the presence of rhizobial strains assigned to the different genera within the Proteobacteria phylum in the nodules (Pule-Meulenberg et al., 2010; Krasova-Wade et al., 2014). Those strains were mainly classified as *Bradyrhizobium* sp., although Sarr et al. (2009) also detected a strain of *Ralstonia*, a genus from the Betaproteobacteria class. In Brazil, where cowpea is an introduced species, a higher diversity of bacteria associated to the nodules has been described (Martins et al., 1997; Leite et al., 2009). Bacterial strains assigned to the phylum Firmicutes (genera *Bacillus* and *Paenibacillus*) were obtained from root nodules of cowpea plants cultivated in Brazilian semi-arid soils (Costa et al., 2013). Particularly for the Amazonian region, a number of strains classified as *Pseudomonas, Enterobacter* (Gammaproteobacteria), *Bacillus*, and *Paenibacillus* were isolated from cowpea nodules (Jaramillo et al., 2013; Oliveira-Longatti et al., 2014). Those strains are not able to form nodules in association with cowpea, although some can fix nitrogen and, hence, could possibly contribute to the N supply of the plants.

In the present study, only sequences assigned to *Bradyrhizobium* were found in high abundance in all samples. This strongly indicates that, as expected (Pule-Meulenberg et al., 2010), *Bradyrhizobium* species were the major rhizobial symbionts of cowpea irrespectively, of plant genotype and soil type. Furthermore, our data suggest that in the northeast of Brazil, *B. neotropicale*-like strains are the major symbionts of cowpea in soils with no previous cowpea cultivation. These bacteria were first isolated from the neotropical leguminous tree *Centrolobium paraense* and proven to effectively nodulate cowpea (Zilli et al., 2014). Interestingly, sequences of other rhizobial groups more rarely isolated from cowpea nodules, e.g., *Microvirga vignae* (Radl et al., 2014), were in very low abundance in the libraries and, hence, not detectable after the data filtering.

We observed differences between the major OTUs detected in the genotypes BRS Acauã and BRS Pujante, showing that different *Bradyrhizobium* populations were selected by the genotypes. It is possible that differences in exudation pattern lead to the selection of the different bacterial populations, as the spectrum of flavonoids exuded by a legume is considered to be one determinant of host specificity (Kobayashi and Broughton, 2008). Apparently, for the rhizobia-host interaction the plant genotype has a higher influence on the selection of the bacterial symbiont than soil type, as the major OTUs were detected in samples from both soils, but their abundance differs due to plant genotype.

Furthermore, we assumed that a dense populations of *Bradyrhizobium* is present in the investigated soils (Herridge, 2008), even though the areas selected for the experiment had no history of cowpea cultivation. An experiment carried out by Marinho et al. (2014), also at MEF and BEF, showed that the inoculation with high performance rhizobial strains did not lead to significant increases of the nodulation or productivity of all evaluated cowpea genotypes. As discussed by Herridge (2008) these results are normally observed in soils that either have high nitrogen availability or more than 1000 rhizobia per gram of soil. We still do not know how successive cultivation of cowpea would influence the microbiome of cowpea nodules.

We observed a diverse bacterial community associated to cowpea nodules. Similar results were obtained by De Meyer et al. (2015), which carried out a very comprehensive study on the presence of NRE in several plant species grown in different eco-regions. They found that certain bacterial groups dominate in an eco-region when all plants species where analyzed. In our study, we showed that soil type was the major factor driving the composition of the NRE within the same plant species. In cultivation-dependent studies *Pseudomonas, Bacillus*, and *Paenibacillus* were the most frequently detected NRE strains. Interestingly, these groups were not abundant in our libraries. We assume this difference reflect the ability of those microorganisms to grow on the commonly used cultivation conditions. As performed in the present study, combining both strategies might be more appropriated for future investigations on the role of NRE on plant growth and on plant-rhizobia interactions.

We detected a high number of Gammaproteobacteria sequences in our study; those were mainly assigned to the genus *Enterobacter* or were unclassified Enterobacteriaceae. This bacterial family has been frequently described as part of the root microbiome of a number of plants and *Enterobacter* strains were isolated from nodules of many different species of legumes including soybean and cowpea (Li et al., 2008; Deng et al., 2011; Aserse et al., 2013; Oliveira-Longatti et al., 2014; De Meyer et al., 2015). By applying immunofluorescence based microscopy, Muresu et al. (2008) found that around 10% of the cells in nodules of the Mediterranean wild legume *Hedysarum spinosissimum* contained cells of *Pantoea agglomerans*. There has been no evidence that such bacteria can trigger nodule formation, but these endophytic bacteria might enter the nodule via the junction between nodule and the root. Furthermore, as shown by Zgadzaj et al. (2015) endophytic bacteria may also invade legume nodules using the infection thread induced by rhizobia.

Some OTUs from the family Enterobacteriaceae were highly abundant in nodule samples. However, their presence was sporadic, meaning that they are found in only a few of the investigated samples. Even though many strains of Enterobacteriaceae possess plant growth promoting traits, such as production of indole acetic acid (Aserse et al., 2013), and, as shown for other bacterial groups (Martínez-Hidalgo et al., 2014), might actually promote plant growth, the role of these NRE still needs to be investigated. It is possible that depending on environmental conditions effects on plant fitness might vary. Moreover, as already observed for rhizobial symbionts, there might be differences among populations colonizing nodules in the benefits they provide to the host. In this case it was shown that plants tend to sanction nodules colonized by these bacteria, e.g., by decreasing oxygen diffusion or by decreasing carbon delivery (Kiers et al., 2006).

The soil type did significantly influence the composition of the microbiome of cowpea nodules. Some bacterial groups, generally represented by low abundant OTUs, were only detected in plants grown in one soil type. Most interestingly, we observed differences in the composition of *Chryseobacterium* populations. In samples from the Ultisol, a single *Chryseobacterium* OTU (OTU001) was detected, which was even more abundant than *Bradyrhizobium* OTUs. Even though this can be a consequence of bias in multi-template PCR as shown elsewhere (Pinto and Raskin, 2012), it still points to a very high abundance of *Chryseobacterium* sp. in these samples. Interestingly, OTU001 was almost absent in nodules of plants grown in the Vertisol soil. The soil has been described as one of the major factors affecting the composition of endophytic bacterial communities of plant roots (Bulgarelli et al., 2012; Lundberg et al., 2012). It was postulated that a few soil bacteria are either selected by the host immune system or by competition with other microorganisms that also colonize the root tissue. We did not observe a significant effect of plant genotype on NRE composition.

Although the functions of endophytic bacterial communities remain mostly unknown, there are many speculations about their role on plant growth promotion (Velázquez et al., 2013). *Chryseobacterium* was described to increase the tolerance of common bean (*Phaseolus vulgaris*) to moderate saline stress (Estévez et al., 2009). Promotion of germination as well as increase root surface and total nitrogen content in wild lupine could be shown by Gutiérrez-Mañero et al. (2003). It is possible that the presence of *Chryseobacterium* in the root of legume plants might influence interactions between rhizobial symbionts and hosts, as their inoculation caused qualitative changes on the pattern of flavonoids exudated by the host (Dardanelli et al., 2010). *Chryseobacterium* might also help cowpea plants to cope with salinity stress that normally occur in semi-arid regions, as the areas where the cowpea were cropped in this study.

We confirmed the presence of living NRE cells by using a target isolation approach. *Chryseobacterium* strains were detected in the nodules of both genotypes growing in the Ultisol almost 3 years after the first field trial. This proves that the *Chryseobacterium* sequences detected in the libraries did not derive only from extra cellular DNA and that these bacteria indeed colonize cowpea nodules. This evidence also indicates that *Chryseobacterium* is an established member of the bacterial community in the BEF soils. Several studies already isolated other NRE from cowpea nodules belonging to Proteobacteria, Firmicutes, and Actinobacteria phyla (Guimarães et al., 2012; Costa et al., 2013; Jaramillo et al., 2013) using standard media for rhizobial isolation. Nevertheless, Bacteroidetes, such as *Chryseobacterium*, were not obtained up to now, probably because the great part of environmental isolates from this genus are not able to metabolize mannitol (Cho et al., 2010), the carbon source preferred by rhizobia. The role of *Chryseobacterium* and other non-rhizobial nodule-associated bacteria as plant growth promoters must be evaluated in future studies.

Recent studies gave a hint on the possible roles of NRE. For example, there are evidences for the role of *Paenibacillus* on the reduction of oxidative stress in cowpea nodules, leading to a delay of the process of nodule senescence (Rodrigues et al., 2013). However, the authors did not show that these bacteria actually colonize the interior of the nodules after inoculation of cowpea seeds. Therefore, the development of strains expressing fluorescence proteins for the localization of NREs in plant tissue as well as transcriptomic and proteomic studies could help to understand the role of those bacteria in the development of the host plant. Moreover, our data indicate that the soil is a major driver for the composition of the microbiome associated to nodules. This observation could be related to two different mechanisms. Either the plant is able to select, depending on the environmental conditions present, its nodule microbiome from the microbes being present in soil, or soils differ in the abundance of selected microbes and those which are more dominant become part of the nodule microbiome simply as a result of probability. The latter hypothesis is, however, very difficult to prove, as the observed OTUs with high abundance in the nodules are mostly part of the rare biosphere in soil with a total share to the soil microbiome of less than 0.5%. In our study this was also true, for example, for OTUs assigned to *Chryseobacterium*, when Ultisol samples were analyzed (data not shown). Thus, for these OTUs, the assessment of statistical significant differences would require further techniques like qPCR to prove the relevance of soil born bacteria for the composition of the nodule microbiome.

## Author Contributors

JL, PF-J, LR, and GX conceived and designed the field experiment. JL, DF, AH, SK, MS, and VR conceived and designed the lab experiment. JL, PF-J, AH, and SK performed experiments. JL, DF, AH, SK, and VR performed the data evaluation. JL, DF, LR, PF-J, MS, and VR wrote the manuscript. All authors read, edited, and approved the final manuscript.

## Conflict of Interest Statement

The authors declare that the research was conducted in the absence of any commercial or financial relationships that could be construed as a potential conflict of interest.

## Footnotes

1 http://www.mothur.org/wiki/Silva_reference_files

2 https://www.r-project.org/

3 https://www.rstudio.com/

4 http://www.ncbi.nlm.nih.gov/sra

5 http://www.arb-home.de/

## Supplementary Material

FIGURE S1 **Rarefaction curves based on 16S rRNA amplicon data (Legend: Acaua, Pujante = cowpea cultivars BR Acauã and BR Pujante; Vertisol, Ultisol = Soil type from the area where cowpea plants were cultivated; 3, 5, 10 = OTU 97%, OTU 95%, and OTU 90% similarity)**.

## References

[B1] Aserse A. A. Räsänen L. A. Aseffa F. Hailemariam A. Lindström K. (2013). Diversity of sporadic symbionts and nonsymbiotic endophytic bacteria isolated from nodules of woody, shrub, and food legumes in Ethiopia. *Appl. Microbiol. Biotechnol.* 97 10117–10134. 10.1007/s00253-013-5248-4 24196581

[B2] Beijerinck M. W. (1890). Künstliche Infection von Vicia Faba mit *Bacillus radicicola*. Ernährungsbedingungen dieser Bacterie. *Bot. Ztg.* 52 837–843.

[B3] Berg G. Smalla K. (2009). Plant species and soil type cooperatively shape the structure and function of microbial communities in the rhizosphere. *FEMS Microbiol. Ecol.* 68 1–13. 10.1111/j.1574-6941.2009.00654.x 19243436

[B4] Bulgarelli D. Rott M. Schlaeppi K. Ver Loren van Themaat E. Ahmadinejad N. Assenza F. (2012). Revealing structure and assembly cues for *Arabidopsis* root-inhabiting bacterial microbiota. *Nature* 2 91–95. 10.1038/nature11336 22859207

[B5] Cho S. H. Lee K. S. Shin D. S. Han J. H. Park K. S. Lee C. H. (2010). Four new species of Chryseobacterium from the rhizosphere of coastal sand dune plants, Chryseobacterium elymi sp. nov., Chryseobacterium hagamense sp. nov., *Chryseobacterium lathyri* sp. nov. and *Chryseobacterium rhizosphaerae* sp. nov. *Syst. Appl. Microbiol.* 33 122–127. 10.1016/j.syapm.2009.12.004 20185262

[B6] R Core Team (2014). *R: A Language and Environment for Statistical Computing*. Vienna: R Foundation for Statistical Computing.

[B7] Costa E. M. Nóbrega R. S. A. Carvalho F. Trochmann A. Ferreira L. V. M. Moreira F. M. S. (2013). Plant growth promotion and genetic diversity of bacteria isolated from cowpea nodules. *Pesq. Agropec. Bras.* 48 1275–1284. 10.1590/S0100-204X2013000900012

[B8] Dardanelli M. S. Manyani H. González-Barroso S. Rodríguez-Carvajal M. A. Gil-Serrano A. M. Espuny M. R. (2010). Effect of the presence of the plant growth promoting rhizobacterium (PGPR) *Chryseobacterium balustinum* Aur9 and salt stress in the pattern of flavonoids exuded by soybean roots. *Plant Soil* 328 483–493. 10.1007/s11104-009-0127-6

[B9] De Meyer S. E. De Beuf K. Vekeman B. Willems A. (2015). A large diversity of non-rhizobial endophytes found in legume root nodules in Flanders (Belgium). *Soil. Biol. Biochem.* 83 1–11. 10.1016/j.soilbio.2015.01.002

[B10] Deng Z. S. Zhao L. F. Kong Z. Y. Yang W. Q. Lindström K. Wang E. T. (2011). Diversity of endophytic bacteria within nodules of the *Sphaerophysa salsula* in different regions of Loess Plateau in China. *FEMS Microbiol. Ecol.* 76 463–475. 10.1111/j.1574-6941.2011.01063.x 21303396

[B11] Estévez J. Dardanelli M. S. Megías M. Rodríguez-Navarro D. N. (2009). Symbiotic performance of common bean and soybean co-inoculated with rhizobia and *Chryseobacterium balustinum* Aur9 under moderate saline conditions. *Symbiosis* 49 29–36. 10.1007/s13199-009-0008-z

[B12] Frank B. (1889). Über die Pilzsymbiose der Leguminosen. *Ber. Dtsch. Bot. Ges.* 7 332–346. 10.1111/j.1438-8677.1889.tb05711.x

[B13] Girvan M. S. Bullimore J. Pretty J. N. Osborn A. M. Ball A. S. (2003). Soil type is the primary determinant of the composition of the total and active bacterial communities in arable soils. *Appl. Environ. Microbiol.* 69 1800–1809. 10.1128/AEM.69.3.1800-1809.2003 12620873 PMC150080

[B14] Graham P. H. (2008). “Ecology of the root-nodule bacteria of legumes,” in *Nitrogen-fixing Leguminous Symbioses* eds Dilworth M. J. James E. K. Sprent I J. Newton W. E. (Dordrecht: Springer) 23–58. 10.1007/978-1-4020-3548-7_2

[B15] Guimarães A. A. Jaramillo P. M. D. Nóbrega R. S. A. Florentino L. A. Silva K. B. Moreira F. M. S. (2012). Genetic and symbiotic diversity of nitrogen-fixing bacteria isolated from agricultural soils in the western Amazon by using cowpea as the trap plant. *Appl. Environ. Microbiol.* 78 6726–6733. 10.1128/AEM.01303-12 22798370 PMC3426679

[B16] Gutiérrez-Mañero F. J. Probanza A. Ramos B. Colón-Flores J. J. Lucas-García J. A. (2003). Effects of culture filtrates of rhizobacteria isolated from wild lupine on germination, growth and biological nitrogen fixation of lupine seedlings. *J. Plant Nutr.* 26 1101–1115. 10.1081/PLN-120020078

[B17] Gyaneshwar P. Hirsch A. M. Moulin L. Chen W. M. Elliott G. N. Bontemps C. (2011). Legume-nodulating betaproteobacteria: diversity, host range, and future prospects. *Mol. Plant Microbe Interact.* 24 1276–1288. 10.1094/MPMI-06-11-0172 21830951

[B18] Herridge D. F. (2008). “Inoculation technology for legumes,” in *Nitrogen-Fixing Leguminous Symbioses* eds Dilworth M. J. James E. K. Sprent I J. Newton W. E. (Dordrecht: Springer) 77–115. 10.1007/978-1-4020-3548-7_4

[B19] Jaramillo P. M. D. Guimarães A. A. Florentino L. A. Silva K. B. Nóbrega R. S. A. Moreira F. M. S. (2013). Symbiotic nitrogen-fixing bacterial populations trapped from soils under agroforestry systems in the Western Amazon. *Sci. Agric.* 70 397–404. 10.1590/S0103-90162013000600004

[B20] Kiers T. E. Rousseau R. A. Denison R. F. (2006). Measured sanctions: legume hosts detect quantitative variation in *Rhizobium* cooperation and punish accordingly. *Evol. Ecol. Res.* 8 1077–1086.

[B21] Klindworth A. Pruesse E. Schweer T. Peplies J. Quast C. Horn M. (2013). Evaluation of general 16S ribosomal RNA gene PCR primers for classical and next-generation sequencing-based diversity studies. *Nucleic Acids Res.* 41 e1 10.1093/nar/gks808 PMC3592464 22933715

[B22] Kobayashi H. Broughton W. J. (2008). “Fine-tuning of symbiotic genes in rhizobia: flavonoid signal transduction cascade,” in *Nitrogen-Fixing Leguminous Symbioses* eds Dilworth M. J. James E. K. Sprent J. I. Newton W. E. (Dordrecht: Springer) 117–152. 10.1007/978-1-4020-3548-7_5

[B23] Krasova-Wade T. Le Quéré A. Laguerre G. N’Zoué A. Ndione J. A. doRego F. (2014). Eco-geographical diversity of cowpea bradyrhizobia in Senegal is marked by dominance of two genetic types. *Syst. Appl. Microbiol.* 37 129–139. 10.1016/j.syapm.2013.10.002 24373721

[B24] Kunin V. Engelbrektson A. Ochman H. Hugenholtz P. (2010). Wrinkles in the rare biosphere: pyrosequencing can lead to artificial inflation of diversity estimates. *Environ. Microbiol.* 12 118–123. 10.1111/j.1462-2920.2009.02051.x 19725865

[B25] Lane D. J. (1991). “16S/23S rRNA sequencing,” in *Nucleic Acids Techniques in Bacterial Systematics* eds Stackebrandt E. Goodfellow M. (Chichester: John Wiley & Sons) 115–147.

[B26] Leite J. Seido S. L. Passos S. R. Xavier G. R. Rumjanek N. G. Martins L. M. V. (2009). Biodiversity of rhizobia associated with cowpea cultivars in soils of the lower half of the São Francisco River Valley. *Rev. Bras. Ci. Solo* 33 1215–1226. 10.1590/S0100-06832009000500015

[B27] Li J. H. Wang E. T. Chen W. F. Chen W. X. (2008). Genetic diversity and potential for promotion of plant growth detected in nodule endophytic bacteria of soybean grown in Heilongjiang province of China. *Soil Biol. Biochem.* 40 238–246. 10.1016/j.soilbio.2007.08.014

[B28] Li L. Sinkko H. Montonen L. Wei G. Lindström K. Räsänen L. A. (2012). Biogeography of symbiotic and other endophytic bacteria isolated from medicinal *Glycyrrhiza* species in China. *FEMS Microbiol. Ecol.* 79 46–68. 10.1111/j.1574-6941.2011.01198.x 22066910

[B29] Ludwig W. Strunk O. Westram R. Richter L. Meier H. Yadhukumar (2004). ARB: a software environment for sequence data. *Nucleic Acids Res.* 32 1363–1371. 10.1093/nar/gkh293 14985472 PMC390282

[B30] Lundberg D. S. Lebeis S. L. Paredes S. H. Yourstone S. Gehring J. Malfatti S. (2012). Defining the core *Arabidopsis thaliana* root microbiome. *Nature* 488 86–90. 10.1038/nature11237 22859206 PMC4074413

[B31] Marinho R. C. N. Nóbrega R. S. A. Zilli J. E. Xavier G. R. Santos C. A. F. Aidar S. T. (2014). Field performance of new cowpea cultivars inoculated with efficient nitrogen-fixing rhizobial strains in the Brazilian Semiarid. *Pesq. Agropec. Bras.* 49 395–402. 10.1590/S0100-204X2014000500009

[B32] Marschner P. Yang C. H. Lieberei R. Crowley D. E. (2001). Soil and plant specific effects on bacterial community composition in the rhizosphere. *Soil Biol. Biochem.* 33 1437–1445. 10.1016/S0038-0717(01)00052-9

[B33] Martínez-Hidalgo P. Galindo-Villardón P. Igual J. M. Trujillo M. E. Martínez-Molina E. (2014). Micromonospora from nitrogen fixing nodules of alfalfa (*Medicago sativa* L.). A new promising plant probiotic bacteria. *Sci. Rep.* 4:6389 10.1038/srep06389 PMC4165979 25227415

[B34] Martins L. M. V. Neves M. C. P. Rumjanek N. G. (1997). Growth characteristics and symbiotic efficiency of rhizobia isolated from cowpea nodules of the north-east region of Brazil. *Soil Biol. Biochem.* 29 1005–1010. 10.1016/S0038-0717(96)00215-5

[B35] Muresu R. Polone E. Sulas L. Baldan B. Tondello A. Delogu G. (2008). Coexistence of predominantly nonculturable rhizobia with diverse, endophytic bacterial taxa within nodules of wild legumes. *FEMS Microbiol. Ecol.* 63 383–400. 10.1111/j.1574-6941.2007.00424.x 18194345

[B36] Nishioka T. Elsharkawy M. Suga H. Kageyama K. Hyakumachi M. Shimizu M. (2016). Development of culture medium for the isolation of flavobacterium and chryseobacterium from rhizosphere soil. *Microbes Environ.* 31 104–110. 10.1264/jsme2.ME15144 27098502 PMC4912144

[B37] Oksanen J. Blanchet F. G. Kindt R. Legendre P. Minchin P. R. O’Hara R. B. (2012). *vegan: Community Ecology Package. R Package Version 2.0-5*. Available at: http://CRAN.R-project.org/package=vegan

[B38] Oldroyd G. E. Murray J. D. Poole P. S. Downie J. A. (2011). The rules of engagement in the legume-rhizobial symbiosis. *Annu. Rev. Genet.* 45 119–144. 10.1146/annurev-genet-110410-132549 21838550

[B39] Oliveira-Longatti S. M. Marra L. M. Soares B. L. Bomfeti C. A. Da Silva K. Ferreira P. A. A. (2014). Bacteria isolated from soils of the western Amazon and from rehabilitated bauxite-mining areas have potential as plant growth promoters. *World J. Microbiol. Biotechnol.* 30 1239–1250. 10.1007/s11274-013-1547-2 24197786

[B40] Pandya M. Kumar G. N. Rajkumar S. (2013). Invasion of rhizobial infection thread by non-rhizobia for colonization of *Vigna radiata* root nodules. *FEMS Microbiol. Lett.* 348 58–65. 10.1111/1574-6968.12245 24033808

[B41] Partida-Martinez L. P. P. Heil M. (2011). The microbe-free plant: fact or artefact? *Front. Plant Sci.* 2:100 10.3389/fpls.2011.00100 PMC3355587 22639622

[B42] Peix A. Ramírez-Bahena M. H. Velázquez E. Bedmar E. J. (2015). Bacterial associations with legumes. *Crit. Rev. Plant Sci.* 34 17–42. 10.1080/07352689.2014.897899

[B43] Pinto A. J. Raskin L. (2012). PCR biases distort bacterial and archaeal community structure in pyrosequencing datasets. *PLoS ONE* 7:e43093 10.1371/journal.pone.0043093 PMC3419673 22905208

[B44] Pruesse E. Quast C. Knittel K. Fuchs B. M. Ludwig W. G. Peplies J. (2007). SILVA: a comprehensive online resource for quality checked and aligned ribosomal RNA sequence data compatible with ARB. *Nucl. Acids Res.* 35 7188–7196. 10.1093/nar/gkm864 17947321 PMC2175337

[B45] Pule-Meulenberg F. Belane A. K. Krasova-Wade T. Dakora F. D. (2010). Symbiotic functioning and bradyrhizobial biodiversity of cowpea (*Vigna unguiculata* L. *Walp*.) in Africa. *BMC Microbiol.* 23:89 10.1186/1471-2180-10-89 PMC2858033 20331875

[B46] Quince C. Lanzén A. Curtis T. P. Davenport R. J. Hall N. Head I. M. (2009). Accurate determination of microbial diversity from 454 pyrosequencing data. *Nat. Methods* 6 639–641. 10.1038/nmeth.1361 19668203

[B47] Radl V. Simões-Araújo J. L. Leite J. Passos S. R. Martins L. M. Xavier G. R. (2014). Microvirga vignae sp. nov., a root nodule symbiotic bacterium isolated from cowpea grown in semi-arid Brazil. *Int. J. Syst. Evol. Microbiol.* 64 725–730. 10.1099/ijs.0.053082-0 24179178

[B48] Remigi P. Zhu J. Young J. P. W. Masson-Boivin C. (2016). Symbiosis within symbiosis: evolving nitrogen-fixing legume symbionts. *Trends Microbiol.* 24 63–75. 10.1016/j.tim.2015.10.007 26612499

[B49] Rodrigues A. C. Bonifacio A. Antunes J. E. L. Silveria J. A. G. Figueiredo M. V. B. (2013). Minimization of oxidative stress in cowpea nodules by the interrelationship between *Bradyrhizobium* sp. and plant growth-promoting bacteria. *Appl. Soil Ecol.* 64 245–251. 10.1016/j.apsoil.2012.12.018

[B50] Saitou N. Nei M. (1987). The neighbor-joining method: a new method for reconstructing phylogenetic trees. *Mol. Biol. Evol.* 4 406–425. 3447015 10.1093/oxfordjournals.molbev.a040454

[B51] Sarr P. S. Yamakawa T. Fujimoto S. Saeki Y. Thao H. T. Myint A. K. (2009). Phylogenetic diversity and symbiotic effectiveness of root-nodulating bacteria associated with cowpea in the South-West area of Japan. *Microbes Environ.* 24 105–112. 10.1264/jsme2.ME08558 21566362

[B52] Schloss P. D. Westcott S. L. Ryabin T. Hall J. R. Hartmann M. Hollister E. B. (2009). Introducing mothur: open-source, platform-independent, community-supported software for describing and comparing microbial communities. *Appl. Environ. Microbiol.* 75 7537–7541. 10.1128/AEM.01541-09 19801464 PMC2786419

[B53] Töwe S. Wallisch S. Bannert A. Fischer D. Hai B. Haesler F. (2011). Improved protocol for the simultaneous extraction and column-based separation of DNA and RNA from different soils. *J. Microbiol. Methods* 84 406–412. 10.1016/j.mimet.2010.12.028 21256887

[B54] Velázquez E. Martínez-Hidalgo P. Carro L. Alonso P. Peix A. Trujillo M. E. (2013). “Nodular endophytes: an untapped diversity,” in *Beneficial Plant–Microbial Interactions: Ecology and Applications* eds González B. R. M. González-López J. (Boca Raton, FL: CRC Press) 214–236.

[B55] Venables W. N. Ripley B. D. (2002). *Modern Applied Statistics with S* 4th Edn. New York, NY: Springer 10.1007/978-0-387-21706-2

[B56] Vincent J. M. (1970). *A Manual for the Practical Study of the Root-Nodule Bacteria.* Oxford: Blackwell Scientific Publications.

[B57] Warnes G. R. Bolker B. Bonebakker L. Gentleman R. Liaw W. H. A. Lumley T. (2012). *gplots: Various R Programming Tools for Plotting Data.* Available at: http://CRAN.R-project.org/package=gplots

[B58] Wilson K. H. Blitchington R. B. Greene R. C. (1990). Amplification of bacterial 16S ribosomal DNA with polymerase chain reaction. *J. Clin. Microbiol.* 28 1942–1946. 2095137 10.1128/jcm.28.9.1942-1946.1990 PMC268083

[B59] Yarza P. Richter M. Peplies J. Euzeby J. Amann R. Schleifer K. H. (2008). The All-Species Living Tree project: a 16S rRNA-based phylogenetic tree of all sequenced type strains. *Syst. Appl. Microbiol.* 31 241–250. 10.1016/j.syapm.2008.07.001 18692976

[B60] Zgadzaj R. James E. K. Kelly S. Kawaharada Y. de Jonge N. Jensen D. B. (2015). A legume genetic framework controls infection of nodules by symbiotic and endophytic bacteria. *PLoS Genet.* 11:e1005280 10.1371/journal.pgen.1005280 PMC4456278 26042417

[B61] Zilli J. E. Baraúna A. C. da Silva K. De Meyer S. E. Farias E. N. Kaminski P. E. (2014). *Bradyrhizobium neotropicale* sp. nov., isolated from effective nodules of *Centrolobium paraense*. *Int. J. Syst. Evol. Microbiol.* 64 3950–3957. 10.1099/ijs.0.065458-0 25205796

