# Supplementary figures and images for: Cowpea Nodules Harbor Non-rhizobial Bacterial Communities that Are Shaped by Soil Type Rather than Plant Genotype
Source: Front Plant Sci. 2017 Jan 20;7:2064. doi: 10.3389/fpls.2016.02064 (PMC5247471; doi:10.3389/fpls.2016.02064)

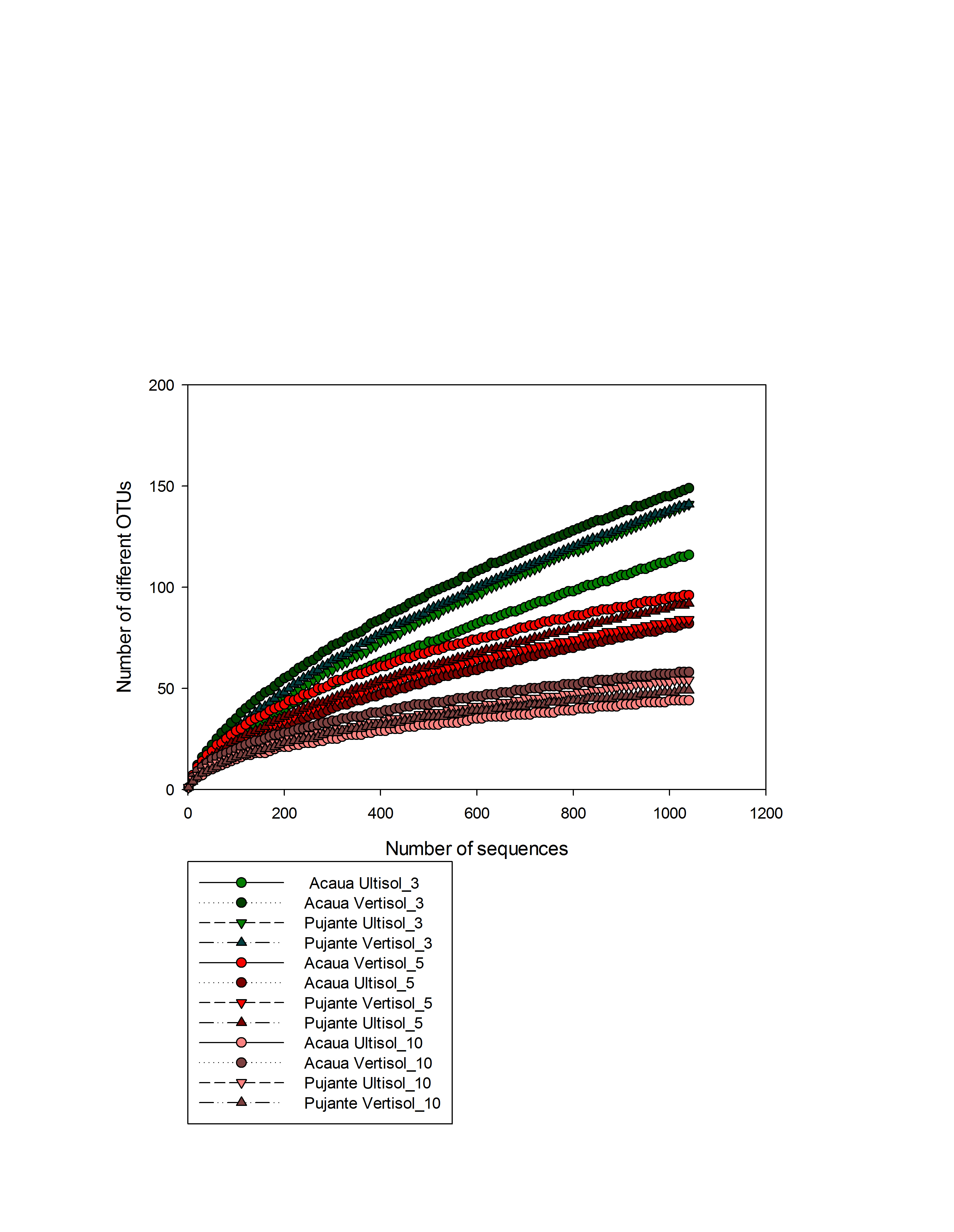

Supplement: FIGURE S1 — Rarefaction curves based on 16S rRNA amplicon data (Legend: Acaua, Pujante = cowpea cultivars BR Acauã and BR Pujante; Vertisol, Ultisol = Soil type from the area where cowpea plants were cultivated; 3, 5, 10 = OTU 97%, OTU 95%, and OTU 90% similarity). [file Image_1.JPEG]
